# Supplementary material for: Novel Driver Strength Index highlights important cancer genes in TCGA PanCanAtlas patients
Source: PeerJ. 2022 Aug 11;10:e13860. doi: 10.7717/peerj.13860 (PMC9375969; doi:10.7717/peerj.13860)
Supplement: Supplemental Information 2 [file peerj-10-13860-s002.zip › COHORTS/genes plots/age/2021_8_16_14_9_distribution_cohorts_50-54.pdf]

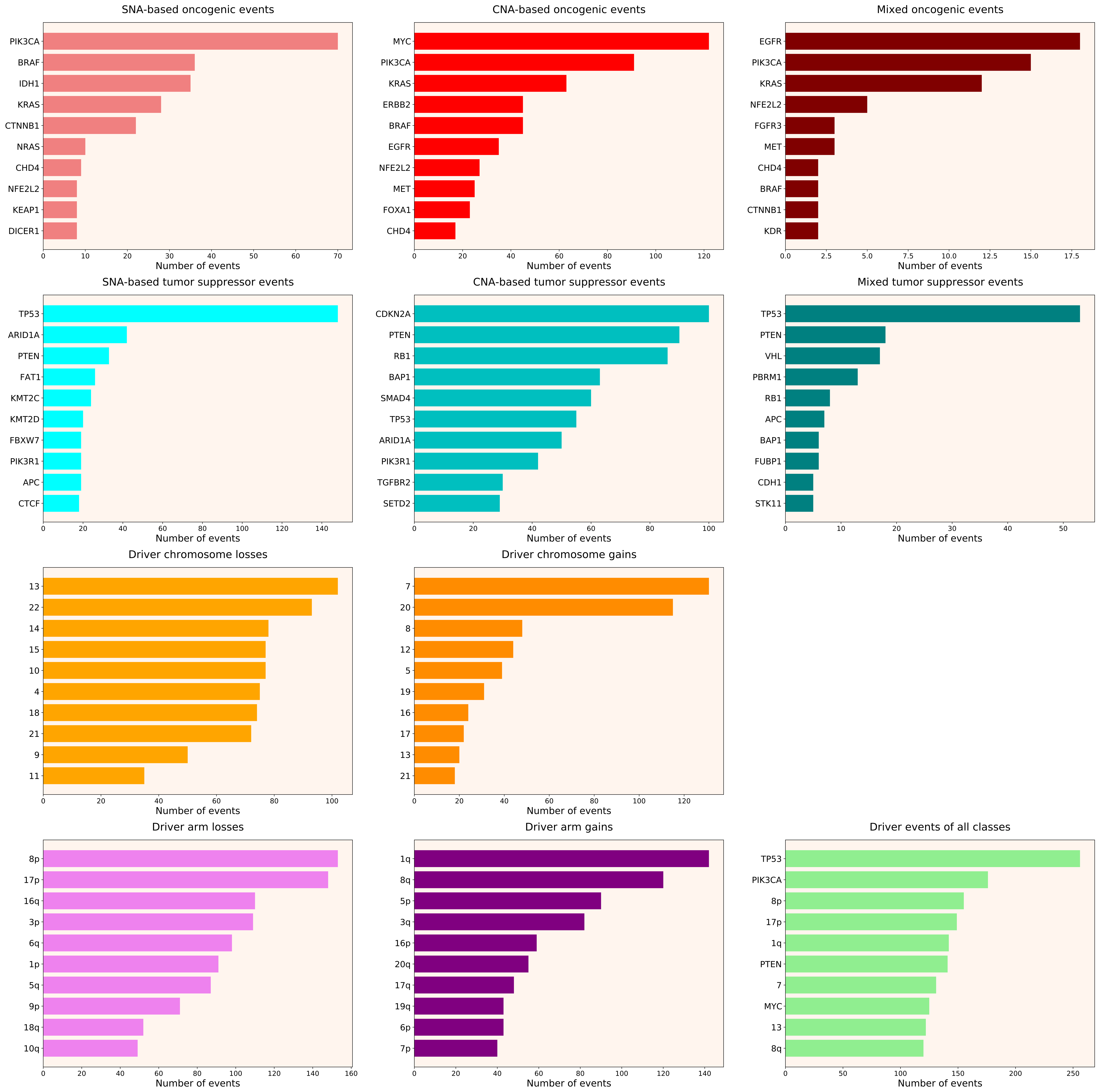

SNA-based oncogenic events
PIK3CA
BRAF
IDH1
KRAS
CTNNB1
NRAS
CHD4
NFE2L2
KEAP1
DICER1
0 10 20 30 40 50 60 70
Number of events
CNA-based oncogenic events
MYC
PIK3CA
KRAS
ERBB2
BRAF
EGFR
NFE2L2
MET
FOXA1
CHD4
0 20 40 60 80 100 120
Number of events
Mixed oncogenic events
EGFR
PIK3CA
KRAS
NFE2L2
FGFR3
MET
CHD4
BRAF
CTNNB1
KDR
0.0 2.5 5.0 7.5 10.0 12.5 15.0 17.5
Number of events
SNA-based tumor suppressor events
TP53
ARID1A
PTEN
FAT1
KMT2C
KMT2D
FBXW7
PIK3R1
APC
CTCF
0 20 40 60 80 100 120 140
Number of events
CNA-based tumor suppressor events
CDKN2A
PTEN
RB1
BAP1
SMAD4
TP53
ARID1A
PIK3R1
TGFBR2
SETD2
0 20 40 60 80 100
Number of events
Mixed tumor suppressor events
TP53
PTEN
VHL
PBRM1
RB1
APC
BAP1
FUBP1
CDH1
STK11
0 10 20 30 40 50
Number of events
Driver chromosome losses
13
22
14
15
10
4
18
21
9
11
0 20 40 60 80 100
Number of events
Driver chromosome gains
7
20
8
12
5
19
16
17
13
21
0 20 40 60 80 100 120
Number of events
Driver arm losses
8p
17p
16q
3p
6q
1p
5q
9p
18q
10q
0 20 40 60 80 100 120 140 160
Number of events
Driver arm gains
1q
8q
5p
3q
16p
20q
17q
19q
6p
7p
0 20 40 60 80 100 120 140
Number of events
Driver events of all classes
TP53
PIK3CA
8p
17p
1q
PTEN
7
MYC
13
8q
0 50 100 150 200 250
Number of events
